# Supplementary material for: The Beneficial Dietary Effect of Dried Olive Pulp on Some Nutritional Characteristics of Eggs Produced by Mid- and Late-Laying Hens
Source: Foods. 2024 Dec 21;13(24):4152. doi: 10.3390/foods13244152 (PMC11675785; doi:10.3390/foods13244152)
Supplement: Supplementary file 1 [file foods-13-04152-s001.zip › foods-3381251-supplementary.pdf]

Table S1. Ingredient composition of the experimental diets (%).

Ingredients (%)	CON	OP4	OP6
Maize	56.6	59.0	57.0
Soya meal-47	25.5	24.8	24.8
Wheat bran	5.0	0	0
Olive pulp	0	4.0	6.0
Marble coarse	3.5	3.5	3.5
Marble powder	5.5	5.5	5.5
Vitamin and mineral premix [1]	2.5	2.5	2.5
Soya oil	1.2	0.5	0.5
Salt	0.1	0.1	0.1
Sodium bicarbonate	0.1	0.1	0.1
Calculated analysis			
Lysine (%)	0.82	0.82	0.82
Methionine + Cystine (%)	0.65	0.65	0.65
Ca (%)	4.25	4.25	4.25
Av. P [2] (%)	0.34	0.34	0.34

[1] Contains per Kg of product: vitamin A, 400,000 IU; vitamin D3, 120,000 IU; vitamin E, 1200 mg; vitamin K3, 160 mg; vitamin B1, 80 mg; vitamin B2, 240 mg; vitamin B6, 120 mg; vitamin B12, 0.8 mg; niacin, 1200 mg; D-calcium pantothenic acid, 480 mg; folic acid, 32 mg; biotin, 2 mg; choline chloride, 21,000 mg; vitamin C, 400 mg; zinc, 4000 mg; manganese, 4800 mg; iron, 2400 mg; copper, 400 mg; iodine, 80 mg; selenium, 8 mg; butilthydroxytoluen (BHT), 25 mg; phytase3, 3400 mg; chemical composition %: proteins, 5%; moisture, 5%; ash, 85%; calcium, 17%; phosphorus, 9.5%; lysine, 1%; methionine, 4%; sodium, 5%. [2] Av. P: available phosphorus.

Table S2. Nutritional analysis of the experimental diets. Data are presented as mean ± standard deviation (mean ± SD). They are derived from the analysis of three samples per batch in a total of three batches.

Parameter	CON	OP4	OP6
Energy (kJ/100 g)	1333.26 ± 34.93	1343.33 ± 24.86	1329.79 ± 38.40
Fat (%)	3.72 ± 0.27	3.51 ± 0.31	3.99 ± 0.10
SFA (%)	0.69 ± 0.16	0.60 ± 0.11	0.64 ± 0.01
MUFA (%)	1.25 ± 0.01	1.21 ± 0.13	1.30 ± 0.13
PUFA (%)	1.78 ± 0.16	1.70 ± 0.15	2.05 ± 0.19
Proteins (%)	17.50 ± 0.47	17.26 ± 0.52	17.29 ± 0.48
Carbohydrates (%)	51.74 ± 1.73	54.61 ±1.82	53.73 ± 1.24
Crude Fiber (%)	2.54 ± 1.16	3.03 ± 1.74	3.29 ± 1.49
Moisture (%)	9.54 ± 0.41	9.36 ± 1.05	9.49 ± 0.45
Ash (%)	14.96 ± 0.95	12.23 ± 1.34	12.21 ± 1.72
Total polyphenols (ppm)	95.40 ± 23.80	123.06 ± 37.66	137.24 ± 24.76
Cholesterol (ppm)	<10	<10	<10

Table S3. Nutritional analysis of the olive pulp used in the trial. Data are presented as mean ± standard deviation (mean ± SD). They are derived from the analysis of three samples per batch in a total of three batches.

Parameter	Olive Pulp
Energy (kJ/100 g)	1464.3 ± 22.01
Proteins (%)	8.5 ± 0.78
Carbohydrates (%)	40.2 ± 2.73
Crude Fiber (%)	29.3 ± 4.23
Moisture (%)	4.3 ± 0.23
Ash (%)	6.9 ± 1.97
Fat (%)	10.9 ± 0.56
Saturated Fatty Acids—SFA (%)	1.7 ± 0.17
Monounsaturated Fatty Acids—MUFA (%)	7.9 ± 0.45
Polyunsaturated Fatty Acids—PUFA (%)	1.3 ± 0.05
Total polyphenols (ppm)	573.70 ± 289.31
Cholesterol (ppm)	<10
Oleuropein (ppm)	20.7 ± 1.54
Hydroxytyrosol (ppm)	<3

Table S4. Fatty acid profile of olive pulp. Data are presented as mean ± standard deviation (mean ± SD). They are derived from the analysis of three samples per batch in a total of three batches.

Fatty Acids (g/100 g Fat)	
Lauric (dodecanoic) acid (C12:0)	0.03 ± 0.02
Myristic acid (C14:0)	0.05 ± 0.01
Palmitic acid (C16:0)	11.23 ± 0.56
Palmitoleic acid (C16:1)	0.57 ± 0.13
Margaric acid (C17:0)	0.12 ± 0.05
Cis-10-Heptadecenoic acid (C17:1)	0.19 ± 0.10
Stearic acid (C18: 0)	2.87 ± 0.05
Oleic acid (C18:1)	71.45 ± 0.44
α-Linoleic acid (C18:2)	10.47 ± 1.12
Linolenic acid(C18:3)	1.24 ± 0.12
Arachidic acid (C20:0)	0.56 ± 0.04
Arachidonic acid (C 20:4 ω6)	0.25 ± 0.14
Behenic acid (C22:0)	0.25 ± 0.05
Tricosanoic acid (C23:0)	0.15 ± 0.13
SFA	15.3 ± 0.78
MUFA	72.6 ± 0.39
PUFA	12.1 ± 1.13
